# Gait Is Associated with Cognitive Flexibility: A Dual-Tasking Study in Healthy Older People

**DOI:** 10.3389/fnagi.2017.00154

**Published:** 2017-05-24

**Authors:** Markus A. Hobert, Sinja I. Meyer, Sandra E. Hasmann, Florian G. Metzger, Ulrike Suenkel, Gerhard W. Eschweiler, Daniela Berg, Walter Maetzler

**Affiliations:** ^1^Center for Neurology and Hertie Institute for Clinical Brain Research, Department of Neurodegeneration, University of TübingenTübingen, Germany; ^2^DZNE, German Center for Neurodegenerative DiseasesTübingen, Germany; ^3^Department of Neurology, University of KielKiel, Germany; ^4^Department of Psychiatry and PsychotherapyUniversity Hospital Tübingen, Tübingen, Germany; ^5^Geriatric Center, University of TübingenTübingen, Germany

**Keywords:** aging, dual tasking, executive function, gait, cognitive flexibility

## Abstract

**Objectives:** To analyze which gait parameters are primarily influenced by cognitive flexibility, and whether such an effect depends on the walking condition used.

**Design:** Cross-sectional analysis.

**Setting:** Tübingen evaluation of Risk factors for Early detection of Neurodegenerative Disorders.

**Participants:** A total of 661 non-demented individuals (49–80 years).

**Measurements:** A gait assessment with four conditions was performed: a 20 m walk at convenient speed (C), at fast speed (F), at fast speed while checking boxes (FB), and while subtracting serial 7s (FS). Seven gait parameters from a wearable sensor-unit (McRoberts, Netherlands) were compared with delta Trail-Making-Test (dTMT) values, which is a measure of cognitive flexibility. Walking strategies of good and poor dTMT performers were compared by evaluating the patterns of gait parameters across conditions.

**Results:** Five parameters correlated significantly with the dTMT in the FS condition, two parameters in the F and FB condition, and none in the C condition. Overall correlations were relatively weak. Gait speed was the gait parameter that most strongly correlated with the dTMT (*r*^2^ = 7.4%). In good, but not poor, dTMT performers differences between FB and FS were significantly different in variability-associated gait parameters.

**Conclusion:** Older individuals need cognitive flexibility to perform difficult walking conditions. This association is best seen in gait speed. New and particularly relevant for recognition and training of deficits is that older individuals with poor cognitive flexibility have obviously fewer resources to adapt to challenging walking conditions. Our findings partially explain gait deficits in older adults with poor cognitive flexibility.

## Introduction

Cognitive flexibility is part of the subdomain “shifting” of executive function (Miyake et al., 2000). It is controlled by the frontal lobe and associated areas (Miyake and Friedman, 2012), and influenced by aging (Wecker et al., 2005) and diseases, such as dementia (Stopford et al., 2012) and stroke (Rasquin et al., 2002). Cognitive flexibility is required for adapting behavior to external influences (Gilbert and Burgess, 2008; Klanker et al., 2013). This flexibility is necessary for the unrestricted performance of daily life during waking states, because it helps to make the right decisions in multitasking situations. Cognitive flexibility is often measured with the delta Trail-Making Test (dTMT) under experimental conditions (Ble et al., 2005; Bowie and Harvey, 2006; Coppin et al., 2006; Montero-Odasso et al., 2009; Hirota et al., 2010). Cognitive flexibility is likely associated with gait particularly during difficult walking situations, such as fast walking and walking when performing additional tasks, but this effect was not investigated in detail. This hypothesis arises from the following studies.

An investigation of 926 older community-dwelling persons with a mean age of 75 years using a single tasking (ST) 4 m walk at a convenient speed and a 7 m fast walk with obstacles (Ble et al., 2005) demonstrated an association between gait speed and cognitive flexibility, as measured with the dTMT in the fast walking with obstacles, but not in the convenient walking paradigm. This report was the first study to indicate an association of cognitive flexibility with gait speed in complex walking conditions in older adults (Ble et al., 2005; Coppin et al., 2006).

In another study of 493 Japanese individuals with a mean age of 74 years, the influence of cognitive flexibility on gait speed in particular under challenging walking conditions was also proposed (Hirota et al., 2010). The participants performed walking tasks of different levels of complexity, and the primary result revealed a stronger association of dTMT with the walking performance in more complex tasks compared to convenient walking.

Finally, the most convincing evidence for an interplay of cognitive flexibility and gait under dual tasking (DT) conditions comes from the Irish longitudinal Study on Aging (Killane et al., 2014). In this study, 4431 participants with a mean age of 62 years underwent walking tasks under ST and DT situations, and cognitive flexibility measures (i.e., the Color Trail Test) correlated significantly with gait speed only during DT, but not ST, situations.

Common in all of these studies is that they found associations of cognitive flexibility with gait. People with poor cognitive flexibility seem to have deficits in gait (control), especially in more difficult gait conditions. This might indicate that people with poor cognitive flexibility have a lower capacity to adapt to the demands of the more difficult walking condition. However, none of the above-mentioned studies reported other quantitative gait parameters beyond gait speed. As gait speed seems to be a sensitive but unspecific parameter to assess health in older age (Studenski et al., 2011), we were interested in whether cognitive flexibility is also associated with quantitative gait parameters “beyond” gait speed and with the “strategies” that are used in walking conditions with different levels of difficulty. This assessment can contribute to a mechanistic model of the interplay between cognitive flexibility and walking behavior.

## Materials and Methods

### Study Participants and Clinical Assessment

Data of 715 healthy, non-demented individuals who participated in the baseline assessment of the TREND study^1^ (**T**übingen evaluation of **R**isk factors for **E**arly detection of **N**eurodegenerative **D**isorders) (Hobert et al., 2011) were considered for this analysis.

The study protocol has been reported elsewhere (Gaenslen et al., 2014). In brief, the TREND study aims at the early detection of neurodegenerative diseases and includes healthy community-dwelling people with or without risk factors for such diseases, i.e., REM sleep behavior disorder, depression, or hyposmia. Study participants were recruited via newspaper advertisements, information events and flyers. All underwent a telephone screening and were considered if they denied psychiatric disorders (other than depression), epilepsy, multiple sclerosis, stroke, dementia, encephalitis malignancies and the need of walking aids. The participants were investigated prospectively in 2009 and 2010.

Out of the 715 participants who performed the measurements, a total of 54 participants were excluded because of technical issues with the sensor system (32), negative or missing dTMT data (13), Mini-Mental State Examination (MMSE) <25 (4) (Folstein et al., 1975) or a diagnosis of Parkinson’s disease (PD) (5) (Hughes et al., 1992). Therefore, 661 subjects were included in the analysis. Excluded participants did not significantly differ from the included cohort in age, sex, or education level. All participants included were between 49 and 80 years of age and able to walk independently without ambulatory aids or assistance. **Table 1** lists the demographic characteristics.

**Table 1:** Demographic data.

	Entire cohort *N* = 661	Good dTMT Performers *N* = 219	Poor dTMT performers *N* = 224	*P*-value
Female [%] ^∗^	53.0	54.8	53.6	0.80
Age [years]	63.2 (7.2)	60.9 (6.7)	65.3 (7.1)	**<0.0001**
MMSE (0–30)	28.8 (1.1)	29.2 (1.0)	28.5 (1.2)	**<0.0001**
TMT A [s]	36.5 (12.1)	34.8 (10.7)	38.8 (13.9)	**0.0009**
TMT B [s]	90.0 (35.8.)	60.3 (12.3)	126.5 (35.1)	**<0.0001**
dTMT [s]	53.5 (31.3.)	25.5 (7.2)	87.7 (28.6)	**<0.0001**
BDI (0–63)	7.9 (6.8.)	7.8 (6.4)	8.3 (6.9)	0.38
Weight [kg]	74.8 (13.5)	74.1 (14.0)	75.3 (13.2)	0.38
Height [cm]	170.8 (8.2)	171.2 (7.9)	170.4 (8.5)	0.27
Education period [years]	14.6 (2.7)	15.2 (2.6)	14.0 (2.7)	**<0.0001**

*^∗^Data are presented with mean and standard deviation or frequency, and p-values were assessed using Student’s t-test and Chi-squared tests. Level of significance (two-sided) was set at 0.05. p < 0.05 are displayed bold. BDI, Beck’s Depression Inventory; dTMT, delta Trail-Making Test (part B - part A); MMSE, Mini Mental State Examination; TMT, Trail-Making Test.*

The ethics committee of the Medical Faculty of the University of Tübingen, Germany approved the study (Nr. 90/2009BO2). All subjects gave informed written consent.

### Gait Assessment

Participants were instructed to walk along a 20 m long, obstacle-free path in an at least 1.5-m wide corridor under the following four conditions: (i) ST walking at a convenient speed; (ii) ST walking at a fast speed; (iii) DT walking at a fast speed and checking boxes at a fast speed; (iv) DT walking at fast speed and subtracting serial 7s at a fast speed. No prioritization of any task was given for the DT tasks. The order of the tasks was (i–iv) for all subjects. In the checking boxes task, study participants were asked to carry a clipboard with a sheet of paper on it. They had to mark the boxes of a table drawn on the paper with a cross as fast as possible. In the subtracting serial 7s task, participants had to subtract 7s from a random three-digit number continuously as fast as possible. The instruction were “Please walk with convenient gait speed and do not risk falling!” for task (i), “Please walk as fast as you can, do not run, do not risk falling!” for task (ii), “Please walk as fast as you can, do not run, do not risk falling, and mark each of the boxes on the sheet of paper with a cross as fast as you can!” for task (iii) and “Please walk as fast as you can, do not run, do not risk falling, and subtract serial 7s as fast as you can from the number I will shortly tell you!” for task (iv).

All subjects wore a small sensor unit (Dynaport Hybrid, McRoberts B.V., The Hague, The Netherlands) that was fixed at the lower back with a belt during the gait tasks. The sensor unit included a 3-axis accelerometer and 3-axis gyroscope with a sampling rate of 100 Hz. Only the middle 70% of steps of the recorded gait information were analyzed to avoid artifacts during gait acceleration and deceleration phases (Lindemann et al., 2008). Overall, number of steps that were included in the analyses ranged from 14 to 29. Quantitative gait parameters were calculated with established algorithms using acceleration in the anterior-posterior direction (Zijlstra and Hof, 2003; Brandes et al., 2006; Dijkstra et al., 2008; Houdijk et al., 2008) through the McRoberts web platform^2^. Raw data were filtered by a bandpass filter between 0.05 and 7 Hz and a tilt correction was used. The included parameters contribute basically to the following gait domains (Verghese et al., 2008): pace (gait speed, number of steps), rhythm (stride duration, double support time) and variability of gait (stride duration variability (calculated using the coefficient of variation (CV) of stride duration (Montero-Odasso et al., 2011)), phase coordination index (PCI, describing the regularity between right and left step phases) (Plotnik et al., 2008), and gait asymmetry (describing the relationship between the average swing times of right and left steps) (Yogev et al., 2007; Plotnik et al., 2009).

### Cognitive Assessment: Trail-Making Test

The time needed to perform the Trail-Making Test (TMT) part B minus A was used to measure cognitive flexibility (dTMT = TMT part B – TMT part A). Details are described elsewhere (Crowe, 1998; Ble et al., 2005; Bowie and Harvey, 2006; Coppin et al., 2006). Briefly, numbers in part A must be connected on a sheet of paper in ascending order as fast as possible. This task primarily tests upper motor performance and visual scanning (Crowe, 1998). Numbers and letters in part B were connected in an alternating manner. This task tests motor performance, visual scanning, and additionally set shifting, i.e., cognitive flexibility (Crowe, 1998).

### Statistical Analysis

Statistical analysis was performed using JMP software (version 11.1.1, SAS). Demographic and clinical parameters of the entire cohort and the subcohorts (see below) are presented as mean and standard deviation or frequency. Comparisons were performed using the Student’s *t*-test and the Chi squared test. The level of significance (two-sided) was set at 0.05 because of the exploratory nature of the study.

Regression analyses with quantitative gait parameters and the dTMT score were performed to analyze the influence of cognitive flexibility on gait parameters. Age, sex, education period, MMSE, and Becks Depression Inventory (BDI) (Hautzinger, 1991) were considered relevant covariates for gait tasks as shown before in this cohort (Hobert et al., 2011) and therefore included in the model.

We also defined the highest and lowest tertile of dTMT performers based on the individual dTMT score, according to Ble et al. (Ble et al., 2005), whether cognitive flexibility influences patterns of significant parameter changes across different walking conditions (Ble et al., 2005; Hobert et al., 2011). We therefore performed intra-group comparisons of every gait parameter between the walking conditions performed at a fast speed (ST fast walk, DT fast walk with checking boxes and DT fast walk with subtracting serial 7s) within good and poor dTMT performers separately using the Wilcoxon test for paired samples. We then compared these patterns of significant parameter changes across different walking conditions between good and poor dTMT performers, i.e., whether we can find significant differences between walking conditions within a dTMT performers group that does not occur in the other group.

## Results

### Correlations between Gait Parameters and Delta TMT Values

No parameter in the convenient ST walking condition was significantly correlated with the dTMT. Two parameters (gait speed (*p* = 0.03) and number of steps (*p* = 0.04)) in the fast ST walking condition were significantly correlated with the dTMT. Two parameters [gait speed (*p* = 0.009) and stride duration (*p* = 0.047)] in the fast DT walking condition with checking boxes were significantly correlated with the dTMT. Five parameters [gait speed (*p* < 0.0001), number of steps (*p* = 0.01)], stride duration (*p* = 0.0006), gait asymmetry (*p* = 0.02), and PCI (*p* = 0.01) in the fast DT walking condition with subtracting serial 7s correlated significantly with the dTMT. Note that this result is not relevantly affected by applying a Bonferroni-corrected *p*-value (0.05/28 = 0.0018). **Table 2** provides the details.

**Table 2:** Correlation values between quantitative gait parameters and the delta Trail-Making Test.

	*r*^2^	*P*-value
Gait speed ST convenient speed	0.024	0.07
Gait speed ST fast speed	0.046	**0.03**
Gait speed DT checking boxes	0.054	**0.009**
Gait speed DT subtracting serial 7s	0.074	**<0.0001**
Number of steps ST convenient speed	0.017	0.17
Number of steps ST fast speed	0.030	**0.04**
Number of steps DT checking boxes	0.024	0.21
Number of steps DT subtracting serial 7s	0.022	**0.01**
Stride duration ST convenient speed	0.000	0.84
Stride duration ST fast speed	0.010	0.54
Stride duration DT checking boxes	0.023	**0.047**
Stride duration DT subtracting serial 7s	0.029	**0.0006**
Double support time ST convenient speed	0.005	0.33
Double support time ST fast speed	0.000	0.80
Double support time DT checking boxes	0.009	0.39
Double support time DT subtracting serial 7s	0.008	0.19
Stride duration CV ST convenient speed	0.000	0.99
Stride duration CV ST fast speed	0.005	0.53
Stride duration CV DT checking boxes	0.000	0.24
Stride duration CV DT subtracting serial 7s	0.005	0.21
Asymmetry ST convenient speed	0.000	0.75
Asymmetry ST fast speed	0.001	0.55
Asymmetry DT checking boxes	0.000	0.57
Asymmetry DT subtracting serial 7s	0.005	**0.02**
PCI ST convenient speed	0.000	0.49
PCI ST fast speed	0.001	0.94
PCI DT checking boxes	0.000	0.63
PCI DT subtracting serial 7s	0.011	**0.01**

*P-Values were corrected for age, Beck’s Depression Inventory (BDI), education period, Mini Mental State Examination (MMSE), and sex. P-values below 0.05 were considered significant and displayed bold. CV, coefficient of variation, DT, dual task; PCI, phase coordination index; ST, single task.*

There is the trend that the highest *r*^2^ values, which indicated the strongest association with the dTMT, of all gait parameters included in the analyses, were found in gait speed across all four walking conditions. The highest *r*^2^ (7.4%) was observed in the walking at fast speed with simultaneously subtracting serial 7s condition.

### Comparison of Gait Parameters between Good and Poor dTMT Performers

No parameter in the convenient ST walking condition was significantly different between the good and the poor dTMT performers. In the fast ST walking condition, two parameters [gait speed (*p* = 0.003) and number of steps (*p* = 0.02)] were different between groups. The following five parameters were significantly different between groups in the fast DT walking condition with checking boxes: gait speed (*p* < 0.0001), number of steps (*p* = 0.02), stride duration (*p* = 0.002), double support time (*p* = 0.02), and stride duration CV (*p* = 0.01). In the fast DT walking condition with subtracting serial 7s, the following six gait parameters were significantly different between the two dTMT groups: gait speed (*p* < 0.0001), number of steps (*p* = 0.03), stride duration (*p* < 0.0001), double support time (*p* = 0.004), gait asymmetry (*p* = 0.01), and PCI (*p* = 0.04). Details are provided in Supplementary Table 1.

### Differences in Gait Adaptation Strategies between Good and Poor dTMT Performers

**FIGURE 1:**
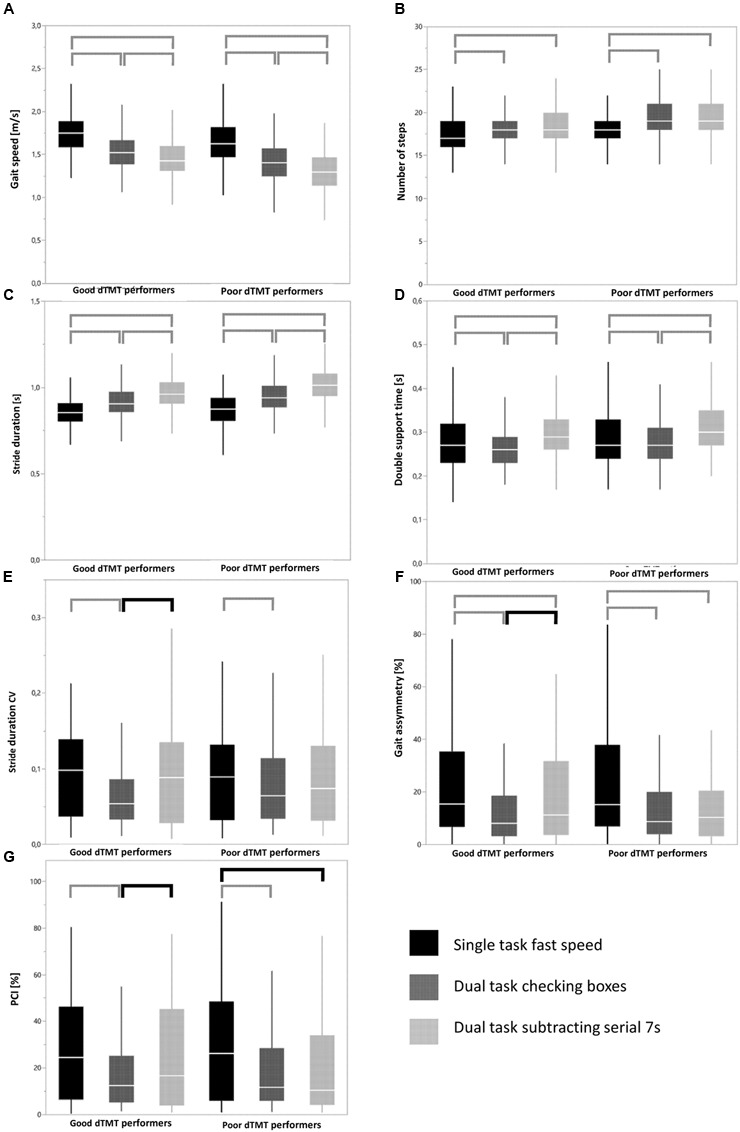
**Overview of patterns of significant parameter changes across different walking conditions in good and poor delta Trail-Making Test (dTMT) performers in different gait parameters (A–G).** Data are shown with box plots. Horizontal lines mark the mean, boxes the first and third quartiles, and whiskers the outermost data point within 1.5-fold the interquartile range above the third quartile or below the first quartile. Square brackets indicate significant differences between walking conditions within a group. Bold square brackets indicate significant differences between distinct walking conditions that occurred in one group but not in the other group. Lower standard deviations in particular in variability-associated parameters under DT than under ST conditions may indicate some rhythmicity, cueing and “magnet” effects induced by the secondary task. These effects have been described previously (Ebersbach et al., 1995; Beauchet et al., 2010). Note that differences of absolute parameter values between groups are not provided in this figure. They are available in Supplementary Table 1. Detailed *p*-values of the square brackets are presented in Supplementary Table 2.

*Both* dTMT groups showed significant differences for gait speed, step time and double support time between the three walking conditions, and significant differences for number of steps between ST walking with fast speed and both DT walking conditions. In other words, the above-mentioned patterns of significant changes from one to another walking condition were identical between the good and poor dTMT performers. On the contrary, good, but not poor, TMT performers showed significant differences for stride time CV, gait asymmetry, and PCI between the DT walking condition with checking boxes and the DT walking condition with subtracting serial 7s. Poor, but not good, dTMT performers showed a significant difference for PCI between the ST walking condition with fast speed and the DT walking condition with checking boxes. In other words, the above-mentioned patterns of significant changes from one to another walking condition were different between the good and poor dTMT performers. **Figure 1** provides an overview of these significant changes, with square brackets that are different between cohorts marked in bold.”

Note that use of a Bonferroni-corrected p-value of 0.0012 did not relevantly affect these results. Only double support time, a parameter relatively closely associated with the gait variability domain (Verghese et al., 2009; Callisaya et al., 2011), then also showed a different pattern of significant parameter changes across the walking conditions between good and poor dTMT performers. For sake of completeness, the correlations between gait parameters are provided in Supplementary Table 3.

## Discussion

This study evaluated quantitative gait parameters of four walking paradigms in a large cohort of healthy older adults wearing a small sensor unit at their lower back, and investigated the association of gait parameters and walking paradigms with cognitive flexibility. Recognizing and addressing such specific deficits in therapy and training settings may lead to improved gait performance in older adults with poor cognitive flexibility.

Our results partially confirm the results of previous studies (Ble et al., 2005; Coppin et al., 2006; Montero-Odasso et al., 2009; Hirota et al., 2010; Berryman et al., 2013; Killane et al., 2014): More challenging walking paradigms require more cognitive flexibility than simple paradigms. In our study, it is reflected by an increasing number of significantly different quantitative gait parameters with increasing difficulty of the walking condition. Accordingly, our observations suggest that the best walking task for the assessment of cognitive flexibility is the DT fast walk with subtracting serial 7s. This task exhibited the highest number of quantitative gait parameters that were significantly associated with the dTMT, and it also included the gait parameter that correlated strongest with the dTMT (gait speed). This observation may be simply explained by the increased influence of supraspinal control mechanisms on gait under more challenging walking conditions (Maetzler et al., 2013; Hobert et al., 2014). However, the obviously higher influence of cognitive flexibility on walking during subtracting serial 7s compared to walking when checking boxes requires further reflection. The control of a DT situation *per se* is a cognitive task, which means that a cognitive process is involved in sharing of resources or shifting of attention between the different tasks, and controls the prioritization of the tasks (Hobert et al., 2011). Based on this, we assumed that the following individual situations were present in our experimental setting. A study participant performed two tasks with mainly motor components (walking and checking boxes) during the checking boxes DT and one cognitive task (the above-mentioned cognitive control of the DT situation). A person during the serial subtraction task performed one task with a primarily motor component (walking) and two tasks with primarily cognitive components (serial subtraction and cognitive control of the DT situation). The bottleneck hypothesis proposes that the processing of two tasks using the same (or similar) network(s) create(s) a bottleneck (Ruthruff et al., 2001; Yogev-Seligmann et al., 2008). Therefore, a gait paradigm with *two* cognitive tasks performed in a (relatively) healthy cohort should exhibit “more” correlation with the dTMT than a gait paradigm with one cognitive task. This presumption was observed in our study.

Indirect support for the above hypothesis comes from studies investigating individuals with motor network deficits, e.g., patients with mild-to-moderate PD (i.e., at a disease stage, where the motor deficits are generally more prominent than the cognitive deficit). These patients might experience the performance of two motor tasks and one cognitive task as more challenging than the performance of one motor task and two cognitive tasks. Our prospective longitudinal study of PD patients with and without falls during an observation period of 3.5 years demonstrated that only DT deficits in the checking boxes task, but not in the subtracting serial 7s task, predicted the first fall in the former group (Heinzel et al., 2016). A recent study did not find any additional value of a cognitive DT paradigm on falls in 263 mild-to-moderate PD patients (Smulders et al., 2012). Unfortunately, the authors did not include a secondary task with a primarily motor component in their study protocol (Smulders et al., 2012).

After the association of cognitive flexibility with walking conditions, we analyzed the association of cognitive flexibility with different gait parameters: The parameter that was most closely associated with the dTMT was gait speed, followed by stride duration and number of steps. This result indicates that pace-associated parameters are more closely correlated with cognitive flexibility than variability-associated parameters, which is basically consistent with the results of a recent study (Martin et al., 2013). However, the authors in the previous study focused on the comparison of gait and executive function in general, and not specifically on cognitive flexibility (Martin et al., 2013). The result is still surprising, because one may associate cognitive flexibility with adaptation, rather than velocity aspects of gait. Regardless of the mechanisms for the “dominance” of gait speed over other quantitative gait parameters for, e.g., detection of gait deficits per se (Lord et al., 2013), motor-cognitive interference deficits (Al-Yahya et al., 2011), and aspects of general health and survival (Studenski et al., 2011), gait speed is a non-specific parameter. This fact is also true for the prediction of cognitive flexibility using this parameter. Gait speed (only) explained 7.4% of the variance of dTMT in the most challenging walking task of our setting.

As a general comment, single quantitative gait parameters may not reach sufficiently high prediction values for any kind of pathology or alterations of human movement that eventually enable an individual diagnosis. The more promising approach to differentiate between specific pathologies / alterations and control states and detect progression and changes due to therapy may be the use of “gait parameter panels” or multivariate regression models. Such models can account for the complex interplay between (dys)function and the compensation mechanisms that are involved in complex movements, such as gait and balance performance in particular under challenging conditions (Maetzler and Hausdorff, 2012; Lord et al., 2013; Maetzler et al., 2013).

This study demonstrated also that cognitive flexibility influences the “type” of walking pattern a person uses in distinct challenging walking situations. Good dTMT performers exhibited significantly increased variability, asymmetry, and irregularity of gait (Hausdorff et al., 2006; Plotnik et al., 2007; Montero-Odasso et al., 2011) in the subtracting serial 7s DT compared to the (easier) checking boxes DT. Notably, poor dTMT performers did not adapt their parameters accordingly, which indicates an impairment of this adaptation strategy. We interpret this finding as follows: Individuals with poor cognitive flexibility reach the maximum adaption capability of their walking pattern earlier than individuals with good cognitive flexibility in a sequence of walking tasks with increasing levels of complexity. A recent study (Lowry et al., 2012) indirectly supports our hypothesis. This study compared the gait parameters of walking along a figure 8 (where changes between walking patterns are necessary) with straight walking (no changes necessary) in 106 old adults and found a significant association between dTMT and the number of steps only when walking on the figure 8 (Lowry et al., 2012).

The present study has some limitations. First, quantitative gait parameters were assessed with a wearable sensor at the lower back. This technique may not be as accurate as more complex gait evaluation systems in measuring at least some of the gait parameters, e.g., double support time. Second, we analyzed approximately 14 m of steady state walking. Although other studies used even shorter distances (Ble et al., 2005; Montero-Odasso et al., 2011; Killane et al., 2014), longer walking distances may deliver more valid gait parameters. It has been shown that the reliability of gait parameters improves with increasing number of steps. This is the case for gait variability parameters (Galna et al., 2013), whereas gait speed reaches already the steady state after 2.5 m (Lindemann et al., 2008). Third, the different tasks were not randomized and may result in a learning effect, but this effect should be comparable in all groups.

## Conclusion

This study demonstrates that cognitive flexibility is associated with walking, in particular under challenging walking conditions, in a cohort of older adults without relevant motor and cognitive deficits. We also demonstrated that older individuals with poor cognitive flexibility use a pattern in variability-related gait parameters across walking conditions that differs from individuals with good cognitive flexibility. This difference might indicate a lower capability of the former population to adapt to challenging walking situations with different demands. Our findings add relevant information to our understanding of gait and balance deficits in older adults with poor cognitive flexibility and may give a basis for interventional studies.

## Author Contributions

MH, DB, and WM made substantial contributions to the acquisition, analysis and interpretation of data for the work. MH, SM, SH, FM, US, and GE made substantial contributions to the acquisition of the data. MH and WM drafted the paper, all remaining authors revised the draft critically for important intellectual content. All authors gave their final approval of the version to be published, and agree to be accountable for all aspects of the work in ensuring that questions related to the accuracy or integrity of any part of the work are appropriately investigated and resolved.

## Conflict of Interest Statement

The authors declare that the research was conducted in the absence of any commercial or financial relationships that could be construed as a potential conflict of interest.

## Footnotes

1 www.trend-studie.de

2 www.mcroberts.nl/analysis
