# Supplementary material for: Gait Is Associated with Cognitive Flexibility: A Dual-Tasking Study in Healthy Older People
Source: Front Aging Neurosci. 2017 May 24;9:154. doi: 10.3389/fnagi.2017.00154 (PMC5442228; doi:10.3389/fnagi.2017.00154)
Supplement: Supplementary file 1 [file Table_1.DOCX]

Supplementary material

**Supplementary Table 1:** Comparison of gait parameters between good and poor dTMT performers

|  | **Good performers**  Mean (SD) | **Poor performers**  Mean (SD) | p-Value |
| --- | --- | --- | --- |
| Gait speed ST convenient speed | 1.38 (0.17) | 1.34 (0.17) | 0.97 |
| Gait speed ST fast speed | 1.75 (0.24) | 1.64 (0.27) | 0.003 |
| Gait speed DT checking boxes | 1.53 (0.20) | 1.42 (0.23) | <0.001 |
| Gait speed DT subtracting serial 7s | 1.45 (0.23) | 1.30 (0.23) | <0.001 |
|  |  |  |  |
| Number of steps ST convenient speed | 18.7 (2.35) | 19.3 (2.6) | 0.74 |
| Number of steps ST fast speed | 17.32 (2.15) | 18.15 (2.67) | 0.02 |
| Number of steps DT checking boxes | 18.40 (2.30) | 19.25 (2.96) | 0.02 |
| Number of steps DT subtracting serial 7s | 18.41 (2.57) | 19.15 (2.75) | 0.03 |
|  |  |  |  |
| Stride duration ST convenient speed | 1.01 (0.07) | 1.01 (0.08) | 0.23 |
| Stride duration ST fast speed | 0.86 (0.09) | 0.87 (0.10) | 0.44 |
| Stride duration DT checking boxes | 0.91 (0.09) | 0.94 (0.10) | 0.0019 |
| Stride duration DT subtracting serial 7s | 0.97 (0.09) | 1.03 (0.17) | <0.001 |
|  |  |  |  |
| Double support time ST convenient speed | 0.28 (0.05) | 0.29 (0.05) | 0.07 |
| Double support time ST fast speed | 0.28 (0.07) | 0.29 (0.07) | 0.49 |
| Double support time DT checking boxes | 0.26 (0.05) | 0.28 (0.05) | 0.016 |
| Double support time DT subtracting serial 7s | 0.30 (0.05) | 0.31 (0.07) | 0.004 |
|  |  |  |  |
| Stride duration CV ST convenient speed | 0.06 (0.05) | 0.06 (0.06) | 0.05 |
| Stride duration CV ST fast speed | 0.09 (0.06) | 0.09 (0.05) | 0.36 |
| Stride duration CV DT checking boxes | 0.07 (0.04) | 0.08 (0.06) | 0.01 |
| Stride duration CV DT subtracting serial 7s | 0.09 (0.06) | 0.08 (0.06) | 0.69 |
|  |  |  |  |
| Asymmetry ST convenient speed | 13.6 (18.6) | 16.4 (20.6) | 0.13 |
| Asymmetry ST fast speed | 26.66 (29.12) | 27.41 (30.11) | 0.29 |
| Asymmetry DT checking boxes | 14.76 (20.10) | 16.37 (20.30) | 0.53 |
| Asymmetry DT subtracting serial 7s | 20.67 (23.35) | 16.17 (19.66) | 0.01 |
|  |  |  |  |
| PCI ST convenient speed | 15.7 (19.8) | 17.5 (19.8) | 0.09 |
| PCI ST fast speed | 28.14 (21.14) | 28.42 (22.47) | 0.68 |
| PCI DT checking boxes | 17.67 (15.99) | 18.92 (17.45) | 0.30 |
| PCI DT subtracting serial 7s | 24.73 (22.44) | 20.04 (19.84) | 0.04 |

Data are presented with mean and standard deviation. P-values were assessed with a regression model with correction for age, Beck’s Depression Inventory (BDI), education period, Mini Mental State Examination (MMSE), and sex. P-values below 0.05 were considered significant. CV, coefficient of variation, DT, dual task; PCI, phase coordination index; ST, single task.

**Supplementary Table 2:** P-values for the comparisons of gait parameters across walking conditions within the good and poor dTMT groups (corresponding to Figure 1 a-g).

|  | **ST / DT checking boxes** | **ST / DT subtracting** | **DT checking boxes / DT subtracting** |
| --- | --- | --- | --- |
| **a, Gait speed** |  |  |  |
| Good dTMT performers | <0.0001 | <0.0001 | <0.0001 |
| Poor dTMT performers | <0.0001 | <0.0001 | <0.0001 |
|  |  |  |  |
| **b, Number of steps** |  |  |  |
| Good dTMT performers | <0.0001 | <0.0001 | 1.0 |
| Poor dTMT performers | <0.0001 | <0.0001 | 0.5 |
|  |  |  |  |
| **c, Stride duration** |  |  |  |
| Good dTMT performers | <0.0001 | <0.0001 | <0.0001 |
| Poor dTMT performers | <0.0001 | <0.0001 | <0.0001 |
|  |  |  |  |
| **d, Double support time** |  |  |  |
| Good dTMT performers | 0.0003 | 0.0091 | <0.0001 |
| Poor dTMT performers | 0.0088 | <0.0001 | <0.0001 |
|  |  |  |  |
| **e, Stride duration CV** |  |  |  |
| Good dTMT performers | <0.0001 | 0.3 | <0.0001 |
| Poor dTMT performers | 0.026 | 0.5 | 0.1 |
|  |  |  |  |
| **f, Gait asymmetry** |  |  |  |
| Good dTMT performers | <0.0001 | 0.0088 | 0.0020 |
| Poor dTMT performers | <0.0001 | <0.0001 | 0.1 |
|  |  |  |  |
| **g, PCI** |  |  |  |
| Good dTMT performers | <0.0001 | 0.06 | <0.0001 |
| Poor dTMT performers | <0.0001 | <0.0001 | 0.4 |

P-values were assessed with paired t-test between walking conditions within dTMT performer groups. CV, coefficient of variation, DT, dual task; dTMT, delta trail making test; PCI, phase coordination index; ST, single task.

**Supplementary Table 3:** Correlations between gait parameters within walking conditions separated in good and poor delta Trail Making Test (dTMT) performers.

| **Good dTMT performers** | | | | |  | | | |  | **Poor dTMT performers** | | | | | | |
| --- | --- | --- | --- | --- | --- | --- | --- | --- | --- | --- | --- | --- | --- | --- | --- | --- |
| ST fast speed | | | | | | | | |  |  |  |  |  |  |  |  |
|  | Gait speed | Number of steps | Stride duration | Double support time | | Stride duration CV | Asymmetry | PCI |  | Gait speed | Number of steps | Stride duration | Double support time | Stride duration CV | Asymmetry | PCI |
| Gait speed | 1 | **-0.5** | **-0.7** | -0.1 | | **0.2** | 0.1 | **0.2** |  | 1 | **-0.6** | **-0.7** | -0.1 | 0.0 | 0.1 | 0.1 |
| Number of steps |  | 1 | 0.0 | -0.1 | | **-0.2** | -0.1 | **-0.3** |  |  | 1 | 0.1 | 0.0 | 0.1 | 0.1 | 0.1 |
| Stride duration |  |  | 1 | **0.2** | | **-0.2** | -0.1 | -0.1 |  |  |  | 1 | **0.2** | -0.1 | **-0.2** | -0.1 |
| Double support time |  |  |  | 1 | | **0.3** | **0.3** | **0.4** |  |  |  |  | 1 | **0.3** | **0.1** | **0.3** |
| Stride duration CV |  |  |  |  | | 1 | **0.2** | **0.7** |  |  |  |  |  | 1 | **0.3** | **0.7** |
| Asymmetry |  |  |  |  | |  | 1 | **0.6** |  |  |  |  |  |  | 1 | **0.6** |
| PCI |  |  |  |  | |  |  | 1 |  |  |  |  |  |  |  | 1 |
|  | | | | | | | | |  |  |  |  |  |  |  |  |
| DT checking boxes | | | | | | | | |  |  |  |  |  |  |  |  |
|  | Gait speed | Number of steps | Stride duration | Double support time | | Stride duration CV | Asymmetry | PCI |  | Gait speed | Number of steps | Stride duration | Double support time | Stride duration CV | Asymmetry | PCI |
| Gait speed | 1 | **-0.5** | **-0.6** | **-0.3** | | **0.1** | 0.0 | 0.1 |  | 1 | **-0.6** | **-0.6** | **-0.4** | 0.1 | 0.1 | 0.1 |
| Number of steps |  | 1 | -0.1 | **0.2** | | 0.0 | 0.1 | 0.0 |  |  | 1 | **0.1** | **0.2** | 0.0 | 0.0 | 0.0 |
| Stride duration |  |  | 1 | **0.2** | | **-0.2** | -0.1 | **-0.1** |  |  |  | 1 | **0.4** | -0.1 | **-0.2** | **-0.1** |
| Double support time |  |  |  | 1 | | **0.4** | **0.4** | **0.5** |  |  |  |  | 1 | **0.3** | **0.4** | **0.5** |
| Stride duration CV |  |  |  |  | | 1 | **0.5** | **0.8** |  |  |  |  |  | 1 | **0.5** | **0.7** |
| Asymmetry |  |  |  |  | |  | 1 | **0.7** |  |  |  |  |  |  | 1 | **0.7** |
| PCI |  |  |  |  | |  |  | 1 |  |  |  |  |  |  |  | 1 |
|  | | | | | | | | |  |  |  |  |  |  |  |  |
| DT subtracting serial 7s | | | | | | | | |  |  |  |  |  |  |  |  |
|  | Gait speed | Number of steps | Stride duration | Double support time | | Stride duration CV | Asymmetry | PCI |  | Gait speed | Number of steps | Stride duration | Double support time | Stride duration CV | Asymmetry | PCI |
| Gait speed | 1 | **-0.6** | **-0.7** | -0.1 | | **0.4** | **0.3** | **0.4** |  | 1 | **-0.4** | **-0.6** | **-0.3** | **0.2** | 0.1 | **0.3** |
| Number of steps |  | 1 | 0.1 | 0.0 | | **-0.2** | **-0.2** | **-0.2** |  |  | 1 | -0.1 | **-0.1** | **-0.2** | -0.1 | **-0.2** |
| Stride duration |  |  | 1 | **0.2** | | **-0.4** | **-0.3** | **-0.4** |  |  |  | 1 | **0.6** | -0.1 | 0.0 | **-0.2** |
| Double support time |  |  |  | 1 | | **0.4** | **0.3** | **0.4** |  |  |  |  | 1 | **0.4** | **0.3** | **0.4** |
| Stride duration CV |  |  |  |  | | 1 | **0.4** | **0.8** |  |  |  |  |  | 1 | **0.4** | **0.8** |
| Asymmetry |  |  |  |  | |  | 1 | **0.6** |  |  |  |  |  |  | 1 | **0.6** |
| PCI |  |  |  |  | |  |  | 1 |  |  |  |  |  |  |  | 1 |

P<0.05 between different parameters are marked in bold. CV, coefficient of variation, DT, dual task; PCI, phase coordination index; ST, single task.
